# The complete chloroplast genome of *Aristolochia hainanensis* Merr. (Aristolochiaceae)

**DOI:** 10.1080/23802359.2022.2119816

**Published:** 2022-09-15

**Authors:** Meixiu Lin, Mengxue Feng, Hui Zhou, Wei Gong, Rongjing Zhang

**Affiliations:** College of Life Sciences, South China Agricultural University, Guangzhou, China

**Keywords:** *Aristolochia hainanensis*, chloroplast genome sequencing, phylogenetic analysis

## Abstract

*Aristolochia hainanensis* Merr. 1922, a well-known Chinese medicinal plant, is distributed in Hainan Province and Guangxi Province, China. In the current study, we sequenced the complete chloroplast genome of *A. hainanensis*. The complete plastome genome was 159,764 bp in length, with a GC content of 38.8%, showing a typical quadripartite organization. The genome contained a large single-copy (LSC) of 89,134 bp, a small single-copy (SSC) of 19,306 bp, and a pair of inverted repeats (IRs) of 25,662 bp. A total of 113 genes were annotated, including 79 protein-coding genes, 30 tRNAs, and four rRNAs. The *trnK*-UUU gene contained the longest intron (2644 bp). The topology of the maximum-likelihood tree supported a close relationship between *A. hainanensis* and *A. kwangsiensis*.

The Aristolochiaceae are composed of approximately 550 species, most of which belong to a large group in the genus *Aristolochia*, which includes 450 species (Bliss et al. 2013; Chase et al. 2016; Qin et al. 2021). In the current study, we focused on the species *Aristolochia hainanensis* Merr. 1922, which is a traditional Chinese medicinal plant mainly distributed in the Wuzhishan Mountain region of Hainan Island. In recent years, with the degradation and loss of their natural growth environment and human harvesting and cutting, *A. hainanensis* has been listed as a Threatened Species with vulnerable (VU) status on the IUCN Red List (https://www.iucnredlist.org/).

Chloroplasts are important organelles of green plants with roles in photosynthesis and nitrogen fixation (Moore et al. 2007). The chloroplast genome is crucial for the taxonomic classification and phylogenetic analysis of land plants (Moore et al. 2007; Yang et al. 2013; Huang et al. 2014). In recent years, many chloroplast genomes of other species in the genus *Aristolochia* have been reported, including *A. kaempferi*, *A. kunmingensis*, *A. macrophylla*, *A. mollissima*, *A. moupinensis*, *A. tagala*, and *A. tubiflora* (Zhou et al. 2017; Li et al. 2019). In the current study, we revealed the chloroplast genome of *A. hainanensis* for the first time, and aimed to provide basic genetic information harbored in the chloroplast and unveil the phylogenetic position of *A. hainanensis*.

**Figure 1.**
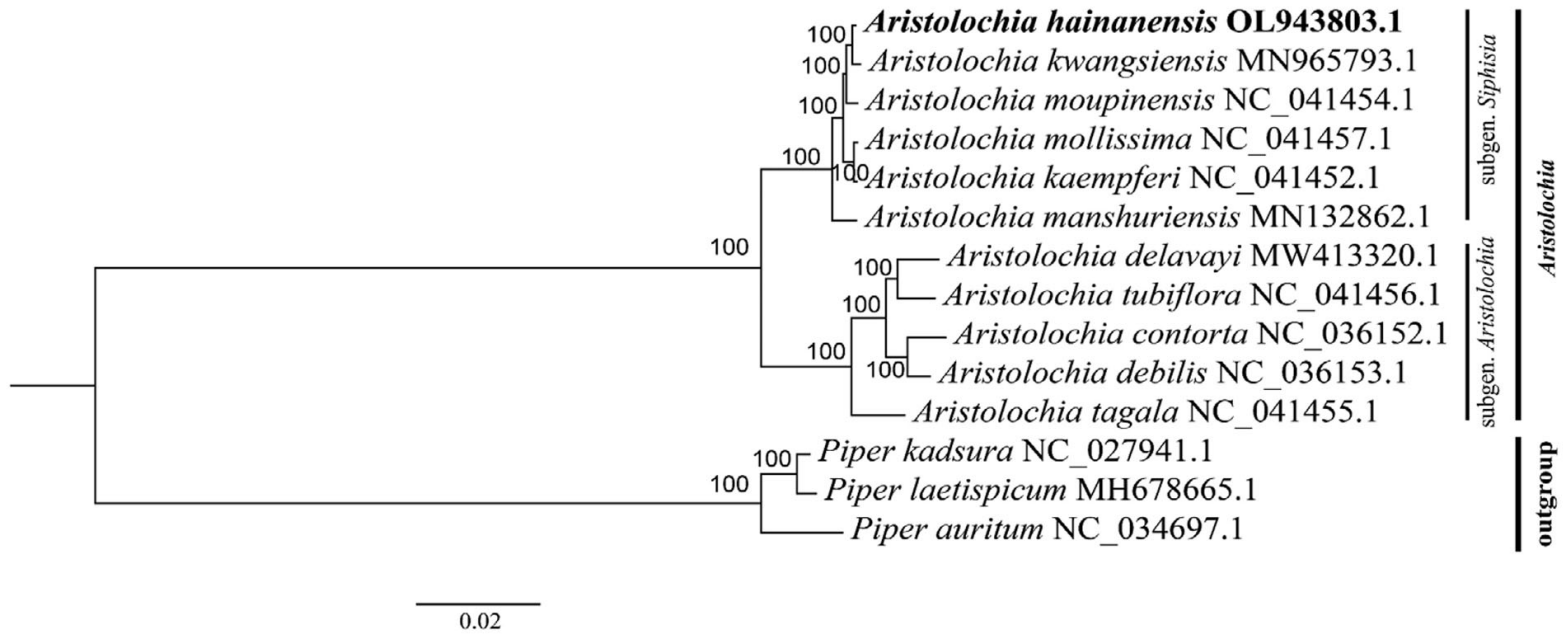
Maximum-likelihood tree based on 11 complete chloroplast genomes of *Aristolochia* and three outgroup species. Numbers in the nodes are bootstrap support values based on 1,000 replications. *A. hainanensis* is highlighted in bold.

We collected several individuals from Mt. Wuzhishan, Hainan, China (E109°42′33.5″, N18°50′03.2″). A specimen was deposited at SCAUB (https://www.cvh.ac.cn/, Rongjing Zhang, zhangrongj@scau.edu.cn) under the voucher number Rong-Jing Zhang 32021. Fresh leaves were sampled for total genomic DNA extraction using a modified CTAB method (Doyle et al. 1987). The DNA library was sequenced using an Illumina NovaSeq 6000 platform with 150 bp pair-end read lengths at Novogene Co. Ltd. (Beijing, China). After filtering low quality reads, a total of 2.38 Gb of clean data with Q30 (92.02%) were obtained and assembled with GetOrganelle (Jin et al. 2020). Annotation was conducted using PGA (Qu et al. 2019) followed by a manual check with Geneious Prime v.2021.2.2 (Biomatters, Auckland, New Zealand, https://www.geneious.com/) to obtain the final annotated data. The complete plastome genome was submitted to GenBank under the accession No. OL943803.

The complete plastome genome was found to be 159,764 bp in length with a GC content of 38.8%, including a large single-copy (LSC) of 89,134 bp and a small single-copy (SSC) of 19,306 bp separated by a pair of inverted repeats (IRs) of 25,662 bp. In total, 113 genes were annotated, including 79 protein-coding genes, four rRNAs, and 30 tRNAs. Among these genes, 15 contained one intron and three contained two introns. The *trnK*-UUU gene included the longest intron at 2644 bp.

To explore the phylogenetic relationship, a phylogenetic tree was generated using five species of the subgenus *Aristolochia* and six species of the subgenus *Siphisia*. Three species of the genus *Piper* were used as outgroups. The sequences used for alignment are available in the GenBank database, including *A. hainanensis* (OL943803.1), *A. kwangsiensis* (MN965793.1), *A. moupinensis* (NC_041454.1), *A. mollissima* (NC_041457.1), *A. kaempferi* (NC_041452.1), *A. manshuriensis* (MN132862.1), *A. delavayi* (MW413320.1), *A. tubiflora* (NC_041456.1), *A. contorta* (NC_036152.1), *A. debilis* (NC_036153.1), *A. tagala* (NC_041455.1), as well as *Piper kadsura* (NC_027941.1), *P. laetispicum* (MH678665.1), and *P. auritum* (NC_034697.1). Sequences were aligned using the MAFFT alignment tool on Geneious Prime v.2021.2.2 (Biomatters, Auckland, New Zealand, https://www.geneious.com/). The best model was generated using jModelTest2 on XSEDE (https://www.phylo.org/, Miller et al. 2010; Darriba et al. 2012). Based on the substitution GTR + I + G model, the maximum-likelihood (ML) phylogenetic tree was produced using RAxML-HPC2 on XSEDE (https://www.phylo.org/) with 1,000 bootstrap replicates (Stamatakis et al. 2008). The topology showed that 11 species of the genus *Aristolochia* occur in the same clade. *A. hainanensis* displayed as a sister to *A. kwangsiensis* based on the ML tree  (Figure 1). This study provides a basis for further research and will aid the conservation of this species.

## Ethics statement

The authors have complied with the International Union for Conservation of Nature (IUCN) policies research involving species at risk of extinction, the Convention on Biological Diversity and the Convention on the Trade in Endangered Species of Wild Fauna and Flora. Our study has been approved by local authorities (Hainan Administration of Wuzhishan National Nature Reserve). Lei Liu is the Deputy Director (e-mail: 57019386@qq.com).

## Author contributions

Conceived and designed the experiments: RJZ, WG, and MXL. Performed the experiments: MXL, MXF, and HZ. Analyzed the data: MXL. Wrote the paper: MXL. Revised the paper: RJZ, WG, MXL, and MXF. Supervised the project and approved the final version to be published: RJZ and WG. All authors agree to be accountable for all aspects of the work.

## Data Availability

The genome sequence data that support the findings of this study are openly available in GenBank of NCBI at https://www.ncbi.nlm.nih.gov under the accession no. OL943803. The associated BioProject, SRA, and Bio-Sample numbers are PRJNA795315, SRR17478235, and SAMN24706807, respectively.
